# Different drying temperatures modulate chemical and antioxidant properties of mandai cempedak ( *Artocarpus integer*)

**DOI:** 10.12688/f1000research.16617.2

**Published:** 2020-03-10

**Authors:** Anton Rahmadi, Yuliana Sabarina, Sukmiyati Agustin

**Affiliations:** 1 Department of Agricultural Products Technology, Mulawarman University, Samarinda, East Kalimantan, 75119, Indonesia

**Keywords:** Antioxidant activity, Artocarpus integer, drying temperature, polyphenols

## Abstract

**Background:** Mandai, the fermented inner skin of cempedak *(Artocarpus integer),* may have further use as an industrial ingredient while maintaining its antioxidative capacity. The starter culture of *Lactobacillus casei* may induce the Mandai fermentation. This research was carried out (i) to investigate the effect of temperature on yield, chemical properties, and antioxidant activity of starter induced fermented mandai powder, (ii) to find the best drying temperature for the powder, and (iii) to find correlations between phenolic contents and antioxidant activity of the powder.

**Methods:** The drying temperature was used as the variable, and was set at 45, 50, and 55°C at a fixed duration of 18 hours. The control was spontaneously fermented mandai dried at 50°C for 18 hours. Total phenolic content (TPC), hydrolyzed tannic content (HTC), and total flavonoid content (TFC) were spectrophotometrically measured, expressed gallic acid (GAE), tannic acid (TAE), and catechin (CAE) equivalents. The DPPH assay measured antioxidant capacity.

**Results:** The best mandai powder had total phenolic content of 348.8±55.6 mg GAE kg ^-1^, HTC of 143.8±9.3 mg TAE kg ^-1^, TFC of 17.5±1.3 mg CAE kg ^-1^, antioxidant activity (IC _50_) of 56.96 ppm, ash content of 4.0±0.7%, pH value of 5.0±0.8, and yield of 9.3±0.8%. There was a strong correlation between TPC, HTC, TFC, and antioxidant activity.

**Conclusions: **Drying temperature affected all observed parameters but not yield, ash and pH. The temperature of 45°C emerged as the best treatment to produce mandai powder from *L. casei*-inoculated mandai cempedak fermentation. The phenolic components contributed to the antioxidant activity of mandai cempedak.

## Introduction

In South and East Kalimantan, Indonesia, people historically consume the lactic acid bacteria (LAB)-fermented inner skin of cempedak *(Artocarpus integer),* traditionally termed dami or mandai ^1^. The use of the inner skin of cempedak in functional food production may partly reduce agricultural waste ^2^. Mandai may contain phenolics, flavonoids, tannins, and antioxidant activity.

The unfermented cempedak inner skin ( *Artocarpus integer*) contains bioactive components such as phenolics, flavonoids, and carotenoids ^3^. It has antioxidant activity that is potentially higher than the flesh and seeds of cempedak. Mandai with *L. casei* as the starter had better antimicrobial activity against *S. aureus* and *E. coli* in comparison to spontaneous ( *L. plantarum*) mandai ^4^. Besides, mandai may function as a probiotic food ^5^. While maintaining its antioxidative capacity, fermented mandai may have further use as an industrial ingredient primarily as an exotic tropical flavor and a flavor enhancer.

Mandai powder is produced through a drying process. The right drying temperature is required to produce good quality mandai powder. Drying at the correct temperature minimizes antioxidant damage, implicating the ability to reduce free radicals will be higher, as seen in sinom beverage powder ^6^, and cumari peppers ^7^.

This research aims (i) to measure chemical and antioxidant properties of *L. casei*-fermented mandai at 45, 50, 55°C of drying temperature with constant time of drying at 18 hours, which the results are then compared with spontaneously fermented mandai and dried at 50°C for 18 hours; (ii) to find the best drying temperature on starter induced fermented mandai powder; and (iii) to find correlations between phenolic contents and antioxidant activity on starter induced fermented mandai powder.

## Methods

### Producing mandai powder from the inner skin of cempedak

Cempedak was peeled and separated from its husk and flesh, then washed and cut into pieces. The pieces of cempedak inner skin were boiled at 100°C for 5 minutes to remove the sap. The sample was drained and then boiled once more in a sealed container at 100°C for 5 minutes to soften the texture. The sample was then cooled until the temperature was less than 40°C. *L. casei* strain Shirota isolated from Yakult® as starter culture was inoculated at the concentration of 2% (v/v). For spontaneous fermentation, the mandai was directly stored without inoculation. The spontaneous and inoculated mandai were stored for two weeks at a temperature of 8±2°C to allow slow fermentation to occur. After incubation, mandai was drained and blended. The puree of mandai then was dried for 18 hours at the 45, 50, and 55°C, then ground and screened with an 80-mesh sieve. The 18 hours drying time opted from the result of the preliminary research. All reagents and corresponding suppliers are listed in Supplementary File.

### Extraction of mandai powder

For the analysis of TPC, HTC, TFC, and antioxidant activity, 20 g mandai powder was dissolved in 60 ml 95% ethanol (SmartLab cat no. A1035, Indonesia) and macerated for 24 hours. Mandai was filtered through filter paper (Whatman no. 4), and the liquid extract was dried at 50°C for 16 hours. The duration of liquid drying opted from the preliminary optimation research. 

### Yield, ash, and pH

The yield was measured as the ratio of mandai powder to initial mandai cempedak (w/w), while ash contents were measured as described ^8^. About 2 g of the sample was diluted with distilled water to a volume of 20 ml. This mixture was homogenized and allowed to soak for 15 minutes before the pH was measured.

### Phenolic content and antioxidant activity measurement

TPC was estimated by the Folin-Ciocalteu assay and expressed as gallic acid equivalents (GAE), as described previously ^9, 10^. HTC was estimated by the Folin-Ciocalteu assay and expressed in mg kg ^-1^ tannic acid equivalent (TAE), as described ^11^. TFC was estimated using the aluminum chloride (AlCl _3_) method and expressed in mg kg ^-1^ catechin equivalent (CAE), as described ^12^. The antioxidant activity was determined using the 2,2-diphenyl-1-picrylhydrazyl (DPPH) assay, as described ^13^. All standards were purchased from Sigma-Aldrich.

### Statistical analysis

Data in Table 1 except IC _50_ of antioxidant activity were subjected to analysis of variance (ANOVA), and Fisher’s least significant difference test determined the significance of the difference between the averages (at α = 5%, analyzed with GraphPad Prism version 6.0. Values were expressed in average ± standard deviation (SD). IC _50_ of antioxidant activity was produced by employing the non-linear fit of the one-phase association method with GraphPad Prism version 6.0. Pearson correlation analysis was performed with Microsoft Excel 2016.

**Table 1.** Yield, ash, pH phenolic contents, and antioxidant activity of powdered mandai cempedak.

Temperature (°C)	Yield (%)	Ash (%)	pH	TPC (GAE mg/kg)	HTC (TAE mg/kg)	TFC (CAE mg/kg)	Antioxidant Activity (IC _50_ in mg/kg)	
							Average	R-square
45	9.3±0.8a	4.0±0.7	5.0±0.8	358.8±55.6	143.8±9.3	17.5±1.3	56.96	0.93
50	9.1±0.8a	3.5±0.8	4.8±0.8	236.3±12.4 ^c^	96.5±8.7 ^c^	5.1±1.8 ^cb^	66.76	0.96
55	9.1±0.6a	3.0±0.7	4.5±0.5	199.2±13.4 ^c^	67.3±6.6 ^d^	1.6±1.0 ^d^	84.74	0.96
control	9.3±0.6a	3.2±0.9	5.3±0.2	275.1±14.2 ^b^	115.3±8.2 ^b^	7.0±1.3 ^b^	136.78	0.97

Yield, ash, pH, total phenolic content (TPC), hydrolyzed tannin content (HTC), total flavonoid content (TFC) are presented in average ± standard deviation.

Letters after the numbers indicate the least significant difference at α=5%

## Results

### Yield, ash, and pH

The yields of mandai powder at drying temperatures of 45, 50, and 55°C did not differ significantly ( Table 1). The yield of each treatment did not differ significantly with the yield of mandai powder with spontaneous fermented that dried at 50°C (control). A comparison between the ash content of each treatment with control showed no significant difference ( Table 1). The pH values of dissolved mandai powder ranged from 4.5±0.5 to 5.3±0.2. The drying temperature did not affect the pH value of the dissolved mandai powder.

### TPC, HTC, and TFC

The drying temperature significantly affected the TPC of mandai powder. The highest average value of TPC in mandai powder dried at 45°C. In comparison to control, the TPC of each treatment was significantly different. The drying temperatures in each treatment resulted in significantly different HTC in comparison to that of control mandai. The drying temperature of 45°C produced higher HTC than that of mandai powder dried at 50°C and 55°C. The drying temperature significantly affected the TFC of mandai powder. The TFC of mandai powder, which dried at 45 and 55°C were significantly different from the control, but that of dried at 50°C was not significantly different from control.

### Antioxidant activity

Half-maximal inhibitory concentration (IC _50_) value of DPPH for each treatment was obtained through one-phase association equation. In the concept of IC _50_ value, the lower the IC _50_ value means the substance is more potent or showing a better antioxidant capacity. The drying temperature significantly affected the antioxidant activity of mandai powder. Control mandai powder extract had the highest IC _50_ value, while mandai powder extract dried at 45°C had the lowest IC _50_ value of ( Table 2). The TPC, HTC, and TFC have a strong correlation with antioxidant activity. The higher TPC, HTC, and TFC value, the higher the antioxidant activity in mandai powder ( Table 3). Lowering the drying temperature may additionally preserve the content of polyphenols, therefore, may further increase the antioxidant capacity. 

**Table 2.** IC _50_ of antioxidant activity of powdered mandai cempedak.

Treatment	Y _0_	Plateau	K	IC _50_ (Interpolated)
Vitamin C	0	103.40	0.003	209.11
45°C	0	89.99	0.014	56.96
50°C	0	91.12	0.011	66.76
55°C	0	91.44	0.009	84.74
Control	0	87.39	0.006	136.78

Y _0_, (0% inhibition / without extract or Vitamin C); Plateu, maximum peak value; K, inhibition rate constant depended on concentration of Vitamin C or extract; IC _50_, concentration of Vitamin C or extracts inhibiting DPPH reduction by 50%.

**Table 3.** Pearson correlation between total phenolic, tannin, flavonoid, and antioxidant activity of mandai powder.

Variable	R-value		
	TPC	HTC	TFC
**Antioxidant activity**	0,796	0,910	0,783
**TPC**		0,973	0,999
**HTC**			0,967

0,01-0,19 very weak correlation; 0,20-0,39 weak correlation; 0,40-0,59 moderate correlation; 0,60-0,79 strong correlation; 0,80-1,00 very strong correlation.

### Correlation of TPC, HTC, TFC, and antioxidant activity

Based on Pearson correlation analysis, there was a strong correlation between total phenolic content (r = 0.796) and total flavonoid content (r = 0.783) with antioxidant activity. The strongest correlation occurred between total tannin content (r = 0.910) and antioxidant activity in mandai powder.

## Discussion

### Yield, ash, and pH

More water in mandai evaporated at higher temperatures. The evaporated water caused the yield of mandai powder to reduce with increasing drying temperature; however, the water content of the final product is also lesser. A previous report documents that the yield may decrease with increased drying temperatures ^14^. The drying temperature did not affect the ash content of mandai powder. Microbes equally used the mineral resources in the spontaneous and *L. casei*-fermented mandai, so it did not have a different effect on ash content.

During mandai fermentation, the population of LAB increased until day 14, thus lowering pH ^1, 5^. Inferring from previous research, organic acids such as lactic acid and acetic acid were produced to lower the pH and caused a more acidic environment on day 12 of fermentation ^5^. The fermentation medium, duration of fermentation, and the use of starter cultures may play a role in the final pH and organic acid contents of LAB-fermented products. The previous research stated that the pH of spontaneously fermented rye dough was higher than the pH of starter-fermented rye dough ^15^. However, cucumber pickle fermented with LAB produced organic acids that were higher than that of spontaneous fermentation ^16^.

### TPC, HTC, and TFC

TPC of the inner skin of cempedak was at 21.29 mg GAE kg ^-1^ ^3^. TPC of mandai powder ranged from 199.2±13.4 to 348.8±55.6 mg GAE kg ^-1^, higher than that of unfermented cempedak. Phenolic compounds were sensitive to heat treatment so that the drying process reduced the TPC ^17^. The TPC of mandai powder dried at 55°C was the lowest value observed when compared to other treatments. The drying process, especially at higher temperatures (i.e., 55°C), combined with the long drying time duration (i.e., 18 hours), resulted in loss of antioxidant activity ^18^.

In dry conditions, components in the cell, such as membranes and organelles, clump together, resulting in fewer extracted phenolic compounds ^14^. Drying at 50°C quickly disabled the oxidation of polyphenols. However, the initial oxidation of polyphenols might have occurred before drying and led to polyphenol degradation. Phenolic compounds are sensitive, unstable, and susceptible to degradation by oxygen and light ^19^. The enzymatic oxidation of polyphenols components is mostly caused by polyphenol oxidase ^7^. Injury to the cell membrane liberates and therefore activates these enzymes, which convert phenolic compounds to quinones.

Phenolic contents may vary on the types of heat processing, such as drying, boiling, and steaming ^19^. Two consecutive boilings at 100°C for 5 minutes assisted removal of sap in the inner skin of cempedak and texture softening. This two-step boilings can be replaced by one-step boiling at a longer time. However, the use of two consecutive boilings is necessary to reduce the length of heat exposure; therefore, this was included as an essential preparation step in our patent application on mandai fermentation.

Environmental factors affecting phenolic concentrations include weather conditions, seasons, and post-harvest conditions ^20^. Phenolic contents are also related to varieties of different fruits, diversity of extraction methods ^21^, and the type of phenolic components in the plant and its location in the cell, as well as the type of solvent and method of extraction ^22– 24^.

*L. casei* may modify the phenolic component, causing a significant difference between TPC of each treatment and control ^25, 26^. The fermentation and metabolic activity of LAB may play a role on levels of total phenolic in rye dough and bread ^15^ and *Moringa oleifera* leaf powder ^27^. HTC was inversely correlated with drying temperature. Also, the duration of drying contributes to the loss of tannins ^28, 29^, consistent with the results in the drying of yacon ( *Polymnia sonchifolia*) and coffee leaf tea ^30, 31^. Degradation of flavonoid structures is linked to the degree of heat exposure ^30, 32^.

### Correlation of TPC, HTC, TFC, and antioxidant activity

The antioxidant activity of mandai powder was low when compared to the fresh form. Temperature plays a role in retaining the antioxidant activity of the powder ( Table 2). The temperature had a significant effect on the inhibition of free radicals of DPPH in grass jelly ( *Premna serratifolia*) ^33^. A strong correlation was observed between antioxidant activity and the polyphenol contents of mandai powder. Previous studies have reported strong correlations between TPC, HTC, TFC, and antioxidant activity ^34, 35^. The phenolic chemical structure has a role in the inhibition of free radicals, largely depending on the number and position of the hydrogen donation from the hydroxyl group to the aromatic ring of the phenol molecules ^36^.

## Conclusions

Drying temperature affected total phenolic, tannin and flavonoid contents, and antioxidant activity, but did not affect ash content, yield, and pH of starter induced fermented mandai powder. Mandai powder dried at 45°C for 18 hours emerged as the best treatment, with a TPC of 358.8±55.6 mg GAE kg ^-1^ dry sample, HTC of 143.8±9.3 mg TAE kg ^-1^ dry sample, TFC of 17.5±1.3 mg CAE kg ^-1^ dry sample, antioxidant activity (IC _50_) of 56.96 ppm, ash content of 4.0±0.7%, pH value of 5.0±0.8, and yield of 9.3±0.8%. The strongest correlation was shown between HTC and IC _50_ of antioxidant activity on starter induced fermented mandai powder. The phenolic components contributed to the antioxidant activity of mandai cempedak.

## Data availability

### Underlying data

**Dataset 1. All raw data obtained in the present study.** Data include yield, ash weight, pH, and phenolic, tannin, and flavonoid contents. DOI: https://doi.org/10.17605/OSF.IO/Q76SH ^37^.

### Extended data

**Supplementary File 1.** List of all reagents used in the current study, with suppliers ^37^.
