# Supplementary material for: Different drying temperatures modulate chemical and antioxidant properties of mandai cempedak ( Artocarpus integer)
Source: F1000Res. 2020 Mar 10;7:1706. Originally published 2018 Oct 26. [Version 2] doi: 10.12688/f1000research.16617.2 (PMC7065658; doi:10.12688/f1000research.16617.2)
Supplement: Peer review history

# Peer review: Reviewer response for version 2

**DOI:** 10.5256/f1000research.24779.r61110

**Published:** 2020-03-10

**Authors:** Syed Ziaur Rahman, Mohd Ashraf Alam

**Affiliations:** 1 Department of Pharmacology & Deputy Medical Superintendent, Jawaharlal Nehru Medical College Hospital, Aligarh Muslim University, Aligarh, Uttar Pradesh, India; 2 Ibn Sina Academy of Medieval Medicine & Science, Aligarh, India; 3 Department of Pharmacology , Jawaharlal Nehru Medical College Hospital, Aligarh Muslim University, Aligarh, Uttar Pradesh, India

No further comments to make.

We confirm that we have read this submission and believe that we have an appropriate level of expertise to confirm that it is of an acceptable scientific standard.

# Peer review: Reviewer response for version 1

**DOI:** 10.5256/f1000research.18160.r42635

**Published:** 2019-02-11

**Authors:** Francisc Vasile Dulf

**Affiliations:** 1 Faculty of Agriculture, Department of Environmental and Plant Protection, University of Agricultural Sciences and Veterinary Medicine Cluj-Napoca, Cluj-Napoca, Romania

This paper aims: to measure chemical and antioxidant properties of *L. casei*-fermented mandai at different drying temperatures with constant time of drying at 18 hours. The results are then compared with spontaneously fermented mandai and dried at 50°C for 18 hours. The paper proposed also to find the best drying temperature on starter induced fermented mandai powder; and to find correlations between phenolic contents and antioxidant activity on starter induced fermented mandai powder.

 The article is well written. Data is interpreted statistically. They are compared to literature data.

I confirm that I have read this submission and believe that I have an appropriate level of expertise to confirm that it is of an acceptable scientific standard.

# Peer review: Reviewer response for version 1

**DOI:** 10.5256/f1000research.18160.r41487

**Published:** 2018-12-13

**Authors:** Syed Ziaur Rahman, Mohd Ashraf Alam

**Affiliations:** 1 Department of Pharmacology & Deputy Medical Superintendent, Jawaharlal Nehru Medical College Hospital, Aligarh Muslim University, Aligarh, Uttar Pradesh, India; 2 Ibn Sina Academy of Medieval Medicine & Science, Aligarh, India; 3 Department of Pharmacology , Jawaharlal Nehru Medical College Hospital, Aligarh Muslim University, Aligarh, Uttar Pradesh, India

It is very interesting research which has tried to explore the use of Mandai as industrial ingredient while maintaining its antioxidant activity. However, there are some suggestions and queries, which needs to be corrected along with grammatical errors.

  

1. Abstract: Better, if is structured like introduction, aims and objectives, material and methods, results and conclusion.
2. Methods: On page 3, it has been mentioned that cempedak was boiled two times at 100°c for 5 minutes to remove sap and to soften its texture, then there might be a possibility that at this temperature of 100°c, flavonoid and retinoid content be lost or decreased.
3. The mandai storage temp, and the drying period of 18 hours, are selected but without proper referencing. Please add reference if any
4. Extraction process: Here the liquid extract was dried at 50°c for 16 hours. Please add reference if any.
5. Methods: There should be a mentioning of temperatures. Nowhere, temperature of 45, 50 and 55°c is mentioned, though the same is provided in the table.
6. Results: The values mentioned on page no. 4 and in table 1 are not matching. The yield mentioned in table at 50 and 55°c are approximately same, but in the text it is mentioned that there was a significant difference.
7. There should be proper mentioning in text under result section whatever is shown in table 1. With increase of drying temperature, there is decrease in TPC, HTC and TFC. There might be a chance to get more flavonoid and retinoid content in further lowering temperatures.
8. Antioxidant activity results/values shown on page 4 and in table 1 are not matching. In the text it is mentioned that higher TPC, HTC, TFC value means higher the antioxidant activity, but in table, as the temperature is increasing the values for TPC, HTC and TFC are decreasing, and antioxidant activity increasing.

We confirm that we have read this submission and believe that we have an appropriate level of expertise to confirm that it is of an acceptable scientific standard, however we have significant reservations, as outlined above.

# Response

**Published:** 2020-02-14

**Authors:** anton rahmadi

Addressing Comment (1)

 Abstract structure is assigned by the journal already.

 Addressing Comment (2)

 Two consecutive boilings at 100°C for 5 minutes assisted removal of sap in the inner skin of cempedak and texture softening. This two-step boilings can be replaced by one-step boiling at a longer time. However, the use of two consecutive boilings is necessary to reduce the length of heat exposure; therefore, this was included as an essential preparation step in our patent application on mandai fermentation.

 Addressing Comment (3, 4, and 5)

 The changes are incorporated in the second version of the article. The drying time of the mandai powder and the liquid extract were opted from the result of the preliminary optimation research, subjected to our dryer capability.

 Addressing Comment (6 and 7)

 We agree with the reviewers suggestion, i.e. (1) the yields of mandai powder at drying temperatures of 45, 50, and 55 °C did not differ significantly, (2) Lowering the drying temperature may additionally preserve the content of polyphenols, therefore, may further increase the antioxidant capacity.

 Addressing Comment (8)

 In response to the reviewer's comment no.8, we made clear that In the concept of IC _50 _value, the lower the IC _50 _value means the substance is more potent or showing a better antioxidant capacity.
